# Hydrogen sulfide priming enhanced salinity tolerance in sunflower by modulating ion hemostasis, cellular redox balance, and gene expression

**DOI:** 10.1186/s12870-023-04552-w

**Published:** 2023-10-30

**Authors:** Abeer Abdelrazk Younis, Mohamed Magdy F. Mansour

**Affiliations:** Department of Botany, Faculty of Science, Ain Shams University, Cairo, 11566 Egypt

**Keywords:** Antioxidants, AsA-GSH system, Gene expression, Hydrogen sulfide, Redox balance, Salinity stress, Sunflower

## Abstract

**Background:**

The salinity threat represents an environmental challenge that drastically affects plant growth and yield. Besides salinity stress, the escalating world population will greatly influence the world’s food security in the future. Therefore, searching for effective strategies to improve crop salinity resilience and sustain agricultural productivity under high salinity is a must. Seed priming is a reliable, simple, low-risk, and low-cost technique. Therefore, this work aimed to evaluate the impact of seed priming with 0.5 mM NaHS, as a donor of H_2_S, in mitigating salinity effects on sunflower seedlings. Primed and nonprime seeds were established in nonsaline soil irrigated with tape water for 14 d, and then exposed to 150 mM NaCl for 7 d.

**Results:**

Salinity stress significantly reduced the seedling growth, biomass accumulation, K^+^, Ca^2+^, and salinity tolerance index while elevating Na^+^ uptake and translocation. Salinity-induced adverse effects were significantly alleviated by H_2_S priming. Upregulation in gene expression (*HaSOS2*, *HaGST*) under NaCl stress was further enhanced by H_2_S priming. Also, H_2_S reduced lipid peroxidation, electrolyte leakage, and H_2_O_2_ content, but elevated the antioxidant defense system. NaCl-induced levels of ascorbate, glutathione, and α tocopherol, as well as the activities of AsA-GSH cycle enzymes: ascorbate peroxidase, monodehydroascorbate reductase, dehydroascorbate reductase, glutathione reductase, and glutathione *S*-transferase, were further enhanced by H_2_S priming. Increased level of H_2_S and total thiol by NaCl was also further stimulated by H_2_S priming.

**Conclusion:**

H_2_S priming has proved to be an efficient strategy to improve sunflower seedlings’ salinity tolerance by retaining ion homeostasis, detoxifying oxidative damage, modulating gene expression involved in ion homeostasis and ROS scavenging, and boosting endogenous H_2_S. These findings suggested that H_2_S acts as a regulatory molecule activating the functional processes responsible for sunflower adaptive mechanisms and could be adopted as a crucial crop management strategy to combat saline conditions. However, it would be of great interest to conduct further studies in the natural saline field to broaden our understanding of crop adaptive mechanisms and to support our claims.

## Background

Salinity is one of the most critical environmental constraints, affecting plant growth, development, and productivity, especially in the arid and semi-arid regions. High soil salinity has arisen as an important global concern, which hampers sustainable crop production in many agrarian countries like Egypt. In addition, water shortage, hot and dry climate, and rising sea levels due to global warming aggravate the existing salinity problems, thus worsening crop production in inland and coastal areas [1]. Excessive salinity can induce various negative effects in plant cells, including ionic, osmotic, and oxidative stress [2]. Salinity stress builds up toxic Na^+^ and Cl^−^ while decreasing beneficial K^+^ and Ca^2+^ levels, which causes the ionic impact of salinity. High salt concentrations in the soil lower its water potential, which imposes water deficit or osmotic stress of salinity. Plants therefore have difficulty absorbing water from the soil. It is worth mentioning that osmotic stress shows its effects at short-term salinity exposure whereas ionic component effects prevail at salinity long exposure [1]. Further, high salinity induces the overproduction of reactive oxygen species (ROS) that oxidize cellular proteins, nucleic acids, and lipids. Plant cells minimize the ROS level because ROS may act as protective agents, not always damaging, as they play a role in signaling pathways and thus stress tolerance under saline conditions, which depend upon ROS concentration and time [3].

Creating salinity-tolerant crop germplasm is becoming urgent to counterbalance and avoid amplifying these hazardous issues of salinity stress. Salinity tolerance in crops is a physiologically multifaceted trait and is controlled by multiple mechanisms. One crucial mechanism to minimize the deleterious effects of toxic ions is to regulate ion concentrations in response to saline conditions, i.e., ion homeostasis [4]. That is, plants need to reduce Na^+^ content to avoid cytoplasmic injury and toxicity and to retain continued uptake of K^+^ under saline environments, which takes place through minimizing Na^+^ uptake by roots and/or increasing Na^+^ efflux back to the soil, intracellular Na^+^ sequestration, K^+^ retention in the cytosol, control of xylem ion loading and excluding Na^+^ from the shoot [5–7]. The Salt Overly Sensitive (SOS) pathway is essential for ion homeostasis and plant adaptation to salinity stress [8]. Among the three proteins (SOS1, SOS2, SOS3) of the SOS pathway, the protein kinase SOS2 functions as a network hub in the SOS pathway, and its kinase activity is rapidly activated by salinity stress, interacting with SOS3 and forms a complex in the cell membrane, which in turn activates the SOS1 antiporter [8, 9]. This antiporter plays a key role in Na^+^ exclusion to outer spaces and in controlling long-distance Na^+^ transport from the root to the shoot [5, 10]. Also, SOS2 has been illustrated to increase both the transcription of the *SOS1* gene and the activity of the SOS1 protein through direct protein–protein interaction [9], indicating a central role in SOS-mediated Na^+^ extrusion. Additionally, Verslues et al. [11] demonstrated that SOS2 interacts with nucleoside diphosphate kinase 2 and with catalases 2 and 3, suggesting that SOS2 is part of a signaling node connecting salinity stress response with ROS signaling. Other plasma membrane channels and transporters that have key functions in K^+^ maintenance and ion homeostasis under high salinity include AKT1 (K^+^ in channels) and KUP1/HAK/KT (high-affinity K^+^-H^+^ symporters) [12].

Owing to the lowering of the soil water potential induced by high concentrations of salts in the soil solution, plants must reduce their water potential to maintain water gradient and subsequently continue water absorption. Plants therefore cope with this osmotic action of salinity by the accumulation of organic/inorganic solutes; that is an osmotic adjustment [13, 14]. These compatible solutes include soluble sugars, polyols, proline, glycine betaine, polyamines, and phenolic compounds, which are innocent to cellular metabolism even at high concentrations [14, 15]. Inorganic ions contribute to osmotic adjustment including Na^+^, K^+^, and Cl^−^, which constitute 80–95% of the osmotic pressure of the cell sap in halophytes and in glycophytes contribute between 50 and 70% [4].

Salinity-triggered oxidative stress through the production of ROS in chloroplasts, mitochondria, peroxisomes, plasma membrane, and apoplast, is a secondary stress [16]. Therefore, another component of salinity tolerance is ROS detoxification and scavenging which is closely related to the maintenance of cellular redox balance by activating and upregulating the antioxidant system including non-enzymatic antioxidants and enzymatic antioxidants [2, 14, 15]. Superoxide dismutase, catalase, glutathione reductase, and peroxidase are examples of enzymatic antioxidants, while non-enzymatic antioxidants include ascorbic acid, glutathione, glycine betaine, proline, carotenoids, total phenolics, flavonoids, and tocopherols [15, 16]. Enzymatic and nonenzymatic defense systems thus scavenge ROS resulting in maintained cell ultrastructures and hence participate in salinity tolerance. In particular, the activation of the ascorbate–glutathione (AsA-GSH) cycle and modulation of the content of ascorbic acid and glutathione have been shown to play central roles in the stability of redox homeostasis and thus crop salinity resilience [17].

Seed priming has been reported as one of the most promising strategies for improving crop growth, development, and productivity, and balancing ionic homeostasis under salinity stress [18–20]. Seed priming with different agents has been successfully adopted to alleviate the adverse effects and induce crop tolerance and productivity to various stresses [21–23]. Hydrogen sulfide (H_2_S) has recently emerged as an important gaseous multifunctional signaling molecule regulating a myriad of physiological processes in plants and is a powerful tool in modifying plants’ adaptability against multiple abiotic stresses [22, 24–26]. Further, H_2_S promotes stress tolerance to abiotic stress by reinstating redox equilibrium, increasing osmolyte buildup, preserving ion balance, modulating gene expression, and regulating ROS-processing systems by transcriptional or posttranslational modifications [27–32].

Sunflower (*Helianthus annuus* L.) is an economically important oilseed crop, which ranks the fourth largest source of edible oil after soybean, rapeseed, and safflower [33]. Sunflower is a high-yielding oilseed crop and has the potential to bridge the gap existing between consumption and domestic production of edible oil in Egypt. Egypt's self-sufficiency of palatable vegetable oils during the 1960s was 95%, which declined to 31.6% in 2007 [34]. However, it is reported that sunflower growing in the central Egyptian province of Fayoum brings economic hope to farmers and contributes to reducing an exorbitant vegetable oil import bill [33, 34]. This necessitates researchers in Egypt and the Mediterranean region to search for approaches to improve sunflower crop performance under saline conditions and hence exploit the saline soils and elevate oil production. This for sure will help to reduce the oil gap in Egypt. In addition, the sunflower crop is not only used in the feeding of humans but also for industrial and energy uses as well as a high-quality forage by livestock producers [35]. Despite priming and foliar application of H_2_S have been illustrated to have an ameliorative impact on crop species in response to various abiotic stresses [22, 26, 30, 31], to the best of our knowledge, the alleviating impact of seed priming with H_2_S on overcoming the adverse effects of saline conditions on sunflower has not been studied yet in detail. Therefore, works aiming to analyze the ameliorative mechanisms triggered by H_2_S could be a novel approach to enhance crop resilience and productivity under salinity stress, which might contribute to global food security. Herein, we thus provide a comprehensive assessment of the impact of seed priming with H_2_S on sunflower growth, the AsA-GSH system, ion homeostasis, and enzymatic and nonenzymatic antioxidants. In addition, the H_2_S priming impact on *HaSOS2* and *HaGST* gene expression modulation of sunflower seedlings exposed to NaCl stress has also been investigated. Our study thus characterizes the underlying processes contributing to seed H_2_S priming-induced salinity tolerance in sunflower seedlings, with particular emphasis on ion homeostasis, regulating H_2_S metabolism, modulating gene expression, and detoxifying oxidative stress. The results indicated that H_2_S priming upregulated expression levels of genes related to ion homeostasis and antioxidant system, SOS pathway, and GSH pathway, which largely contributed to enhanced sunflower tolerance to high salinity.

## Results

### Growth parameters, Na^+^uptake, Na^+^ translocation, and salinity tolerance index (STI)

**Fig. 1:**
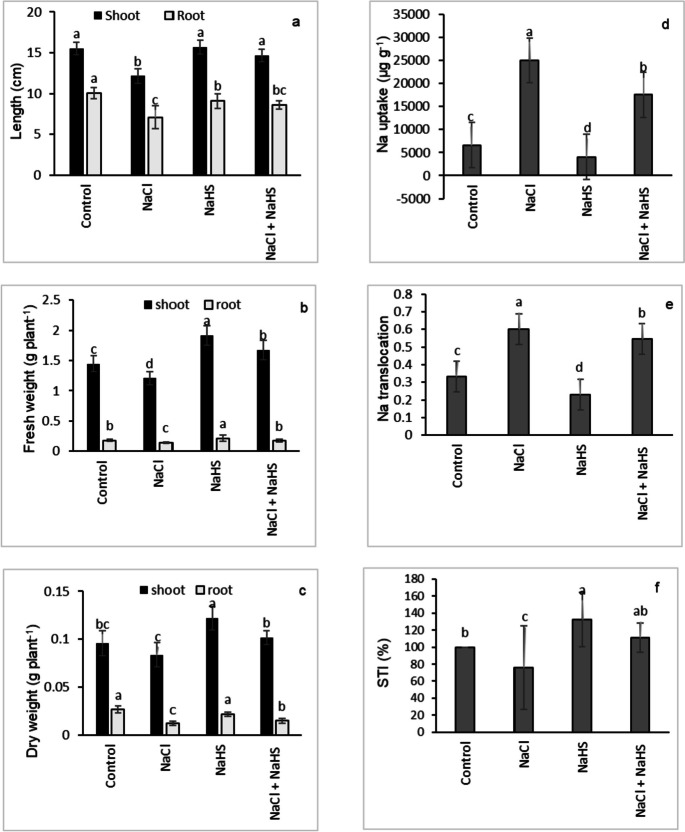
Effect of NaHS priming in the presence and absence of 150 mM NaCl on the shoot and root (**a**) length, (**b**) FW, (**c**) DW, (**d**) sodium uptake, (**e**) sodium translocation, and (**f**) salinity tolerance index (STI) of sunflower shoot and root. Each value is the mean ± SD of three replicates. Bars with different letters indicate significant differences at *P* ≤ 0.05

The shoot length, root length, shoot, and root FW, and shoot and root DW were significantly reduced by 21.9%, 29.3%, 16.4%, 19.7%, 12.7, and 53.6% under salinity stress, respectively, compared with their controls (Fig. 1a-c). However, applying NaHS significantly ameliorated these parameters relative to their corresponding stressed seedlings (Fig. 1a-c). The NaCl-stressed sunflower seedlings recorded the greatest Na^+^ uptake and translocation reaching about 277.9% and 80%, respectively, compared with the control ones (Fig. 1d, e). NaHS pretreatment significantly diminished Na^+^ uptake and translocation by 22.9% and 9%, respectively, compared with the NaCl-stressed alone plants (Fig. 1d, e). In response to NaCl stress, a significant reduction in STI percentage to 24.1 of the value of control seedlings was observed, while NaHS priming significantly enhanced the STI percentage of NaCl-stressed seedlings up to 46.5 (Fig. 1f).

### Mineral contents and *HaSOS2* gene expression

**Fig. 2:**
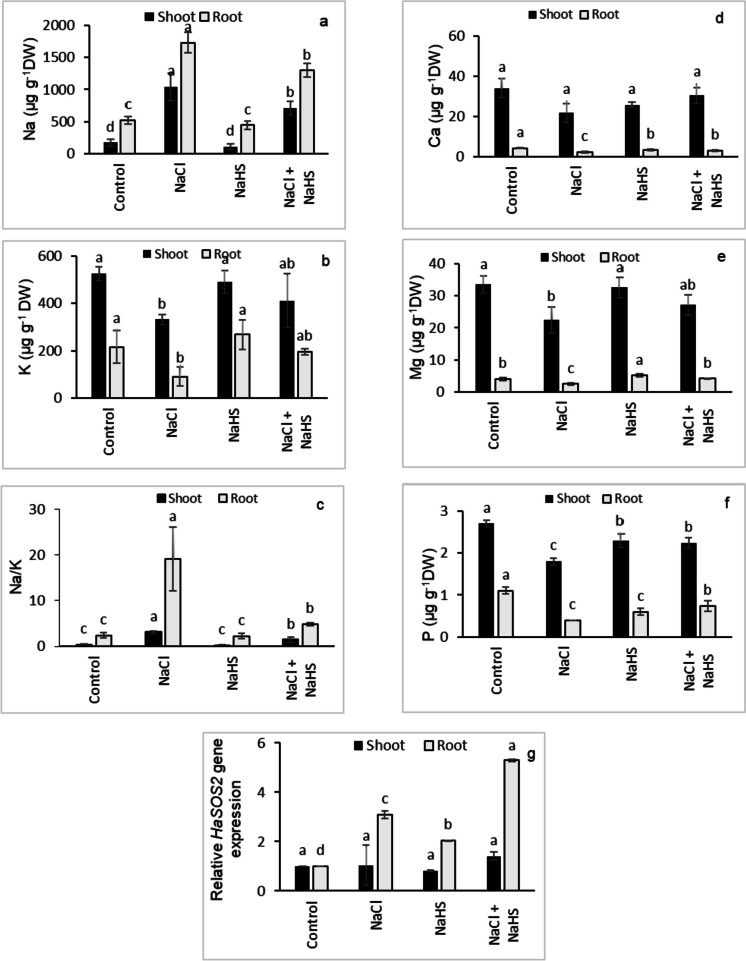
Effect of NaHS priming in the presence and absence of 150 mM NaCl on (**a**) Na^+^ content, (**b**) K^+^ content, (**c**) Na^+^/K^+^ ratio, (**d**) Ca^2+^ content, (**e**) Mg.^2+^ content, (**f**) P content, and (**g**) *SOS2* gene expression of sunflower shoot and root. Each value is the mean ± SD of three replicates. Bars with different letters indicate significant differences at *P* ≤ 0.05

NaCl stress significantly increased the Na^+^ accumulation in the shoots (500%) and roots (233%) of sunflower seedlings relative to their controls, while decreasing the K^+^ content in the shoots (36.8%) and roots (58%) resulting in higher Na^+^/K^+^ ratios (3.12% shoot, 19.1% root) (Fig. 2a-c). NaHS priming significantly reduced the Na^+^ content (31.8% shoot, 25% root), and increased K^+^ content (24% shoot, 117% root), thus lowering Na^+^/K^+^ ratios (53.9% shoot, 74.6% root) compared with NaCl-stressed alone (Fig. 2a-c). The contents of Ca^2+^, Mg^2+^, and P were decreased by 36.5%, 33%, and 33.3%, respectively, in the shoots of salinity-stressed plants relative to their controls. As for the roots, salinity stress caused a decrease in the contents of Ca^2+^, Mg^2+^, and P by 48.7%, 38.3%, and 63.6%, respectively, relative to their controls (Fig. 2d-f). In comparison with salinity-stressed plants, NaHS-pretreated salinity-stressed plants showed increased Ca^2+^, Mg^2+^, and P contents by 40.7%, 20.7%, and 24.1%, respectively, in the shoots and by 39.5%, 67.9%, and 83.3%, respectively, in the roots (Fig. 2d-f). Real-time RT-PCR analysis showed that NaCl treatment significantly increased the transcript level of the *HaSOS2* gene by 209.5% in sunflower roots compared with their control (Fig. 2g). Further elevation in the relative expression of the *HaSOS2* gene by 71.4% was obtained in salinity-stressed roots treated with NaHS compared with seedlings treated with NaCl alone (Fig. 2g).

### H_2_O_2_, malondialdehyde (MDA), electrolyte leakage (EL), and 2,2‐diphenyl‐1‐picrylhydrazyl (DPPH)

**Fig. 3:**
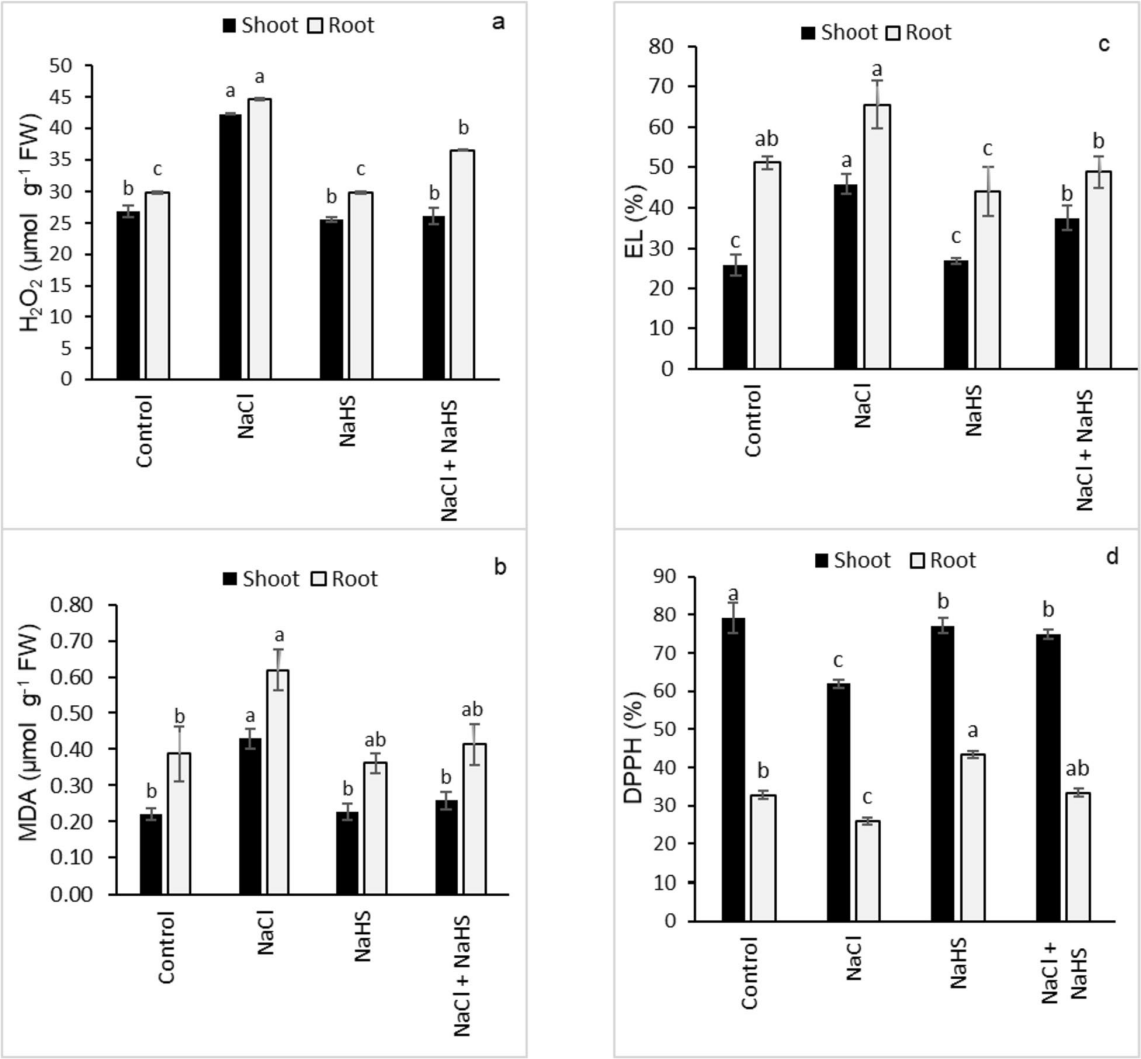
Effect of NaHS priming in the presence and absence of 150 mM NaCl on (**a**) H_2_O_2_ content, (**b**) malondialdehyde (MDA), (**c**) electrolyte leakage (EL), and (**d**) DPPH scavenging percentage of sunflower shoot and root. Each value is the mean ± SD of three replicates. Bars with different letters indicate significant differences at *P* ≤ 0.05

Salinity stress significantly enhanced the contents of H_2_O_2_ by 57.3% and 49.8% and that of MDA by 96.1% and 60% in the shoots and roots, respectively, compared with their controls (Fig. 3a, b). On the other hand, NaHS seed priming before salinity treatment reduced H_2_O_2_ (38.3% and 18.4%) and MDA (40% and 33.3%) contents in shoots and roots, respectively, compared with the seedlings that received only NaCl stress (Fig. 3a, b). Although exposure of the sunflower seedlings to NaCl stress significantly increased EL by 77.1% in the shoots and by 28.4% in the roots relative to their controls, NaHS priming significantly reduced the EL to 18.1% and 25.5% in the shoots and roots, respectively, compared with the plants received NaCl only (Fig. 3c). Salinity stress significantly reduced DPPH radical scavenging activity by 21.9% in the shoots and by 20.8% in the roots relative to their controls (Fig. 3d) while NaHS pretreatment significantly restored DPPH radical scavenging activity to the control level in the shoots and roots of salinity-stressed sunflower seedlings (Fig. 3d).

### Antioxidant enzyme activities and *HaGST* gene expression

**Fig. 4:**
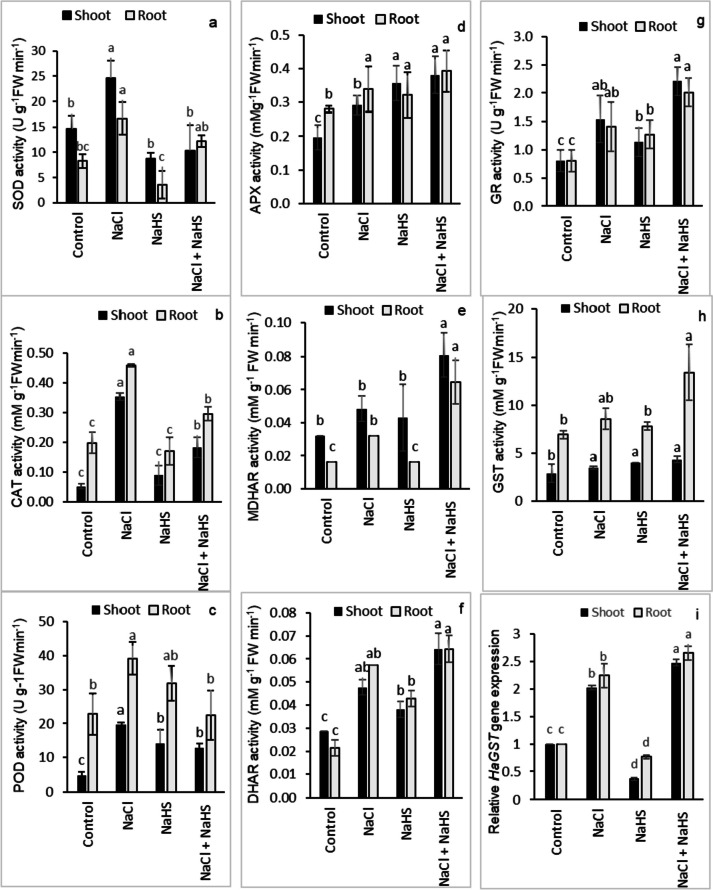
Effect of NaHS priming in presence and absence of 150 mM NaCl on (**a**) superoxide dismutase (SOD), (**b**) catalase (CAT), (**c**) peroxidase (POD), (**d**) ascorbate peroxidase (APX), (**e**) monodehydroascorbate reductase (MDHAR), (**f**) dehydroascorbate reductase (DHAR), (**g**) glutathione reductase (GR), (**h**) glutathione S-transferase (GST), and (**i**) GST gene expression of sunflower shoot and root. Each value is the mean ± SD of three replicates. Bars with different letters indicate significant differences at *P* ≤ 0.05

The activities of superoxide dismutase (SOD), catalase (CAT), and peroxidase (POD) were enhanced by 102.2%, 577.3%, and 309.6% in the shoots and by 42%, 130.6%, and 72% in the roots of NaCl-treated plants, respectively, concerning their respective controls (Fig. 4a-c). On the other hand, NaHS seed priming significantly decreased SOD, CAT, and PX activities in the shoots by 26.9%, 48.3%, and 34.5%, and in the roots by 41.2%, 35.4%, and 42.9%, respectively, as compared with NaCl-treated seedlings (Fig. 4a-c). As for the enzymes of the ascorbate–glutathione (AsA-GSH) cycle, salinity stress significantly enhanced the activities of ascorbate peroxidase (APX), Monodehydroascorbate reductase (MDHAR), dehydroascorbate reductase (DHAR), and glutathione reductase (GR) in the shoots by 48.5%, 50%, 66.7%, and 91.7% and in the roots by 21.3%, 100%, 166.7%, and 75%, respectively, relative to those of the controls (Fig. 4d-g). NaHS pretreatment displayed further enhancement in the APX, MDHAR, DHAR, and GR activities for the shoots by 30.6%, 66.7%, 35%, and 43.5% as well as for the roots by 15.8%, 100% 12.5%, and 42.9%, respectively, in comparison with NaCl-treated plants (Fig. 4d-g). Similarly, under NaCl-free conditions, NaHS priming significantly increased the enzyme activities of the AsA-GSH cycle relative to their untreated controls (Fig. 4d-g).

For GST as one of the GSH-metabolizing enzymes, salinity stress significantly enhanced the GST activity in sunflower shoots and roots by 40% and 23.6%, respectively, relative to their controls, and NaHS priming further increased GST activity reaching 25% and 56.2% in the shoots and roots, respectively, compared with NaCl-treated plants (Fig. 4h). Real-time RT-PCR analysis showed that NaCl treatment resulted in significant increases in *HaGST* transcripts (102.4% and 124.3% in the shoots and roots, respectively), whereas NaHS priming exhibited further enhancement in *HaGST* transcription level by 22.1% and 18.2% in both stressed shoots and roots, respectively, relative to plants received only NaCl treatment (Fig. 4i).

### Nonenzymatic antioxidants, endogenous H_2_S, and total thiol (TT)

**Fig. 5:**
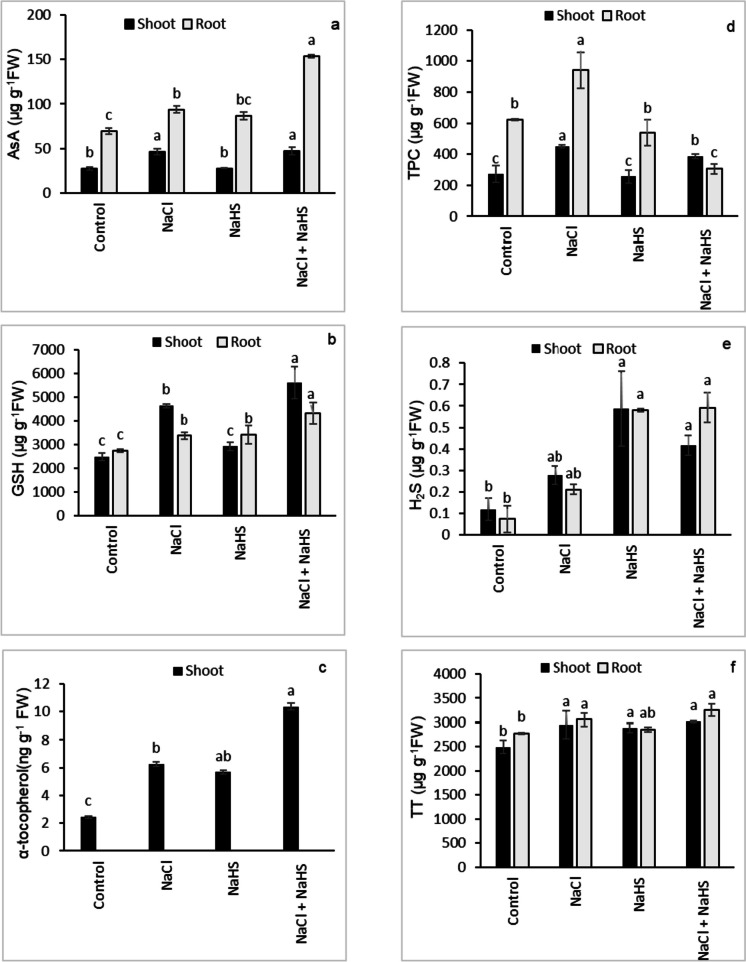
Effect of NaHS priming in the presence and absence of 150 mM NaCl on (**a**) ascorbic acid content (AsA), (**b**) reduced glutathione (GSH), (**c**) α-tocopherol content, (**d**) total phenolic content (TPC), (**e**) H_2_S content, and (**f**) total thiols (TT) of sunflower shoot and root. Each value is the mean ± SD of three replicates. Bars with different letters indicate significant differences at *P* ≤ 0.05

Compared with untreated plants, a significant increase in the concentration of ascorbic acid (AsA) (69.9% shoot, 35.4% root) and reduced glutathione (GSH) (87.5% shoot, 22.9% root) were observed in the shoots and roots of NaCl-stressed plants (Fig. 5a, b). NaHS priming further enhanced the levels of AsA and GSH by 72.1% and 20.8% in the shoots and by 77.2% and 28.3% in the roots, respectively, relative to the seedlings treated with NaCl only (Fig. 5a, b). Additionally, salinity stress significantly promoted α-tocopherol level in the shoots by 155.1% over the untreated control, and NaHS priming further elevated α-tocopherol level by 65.6% in the stressed seedlings compared with those received only NaCl stress (Fig. 5c). The roots of sunflower seedlings showed no α-tocopherol content in response to the different treatments (Fig. 5c). On the other hand, NaHS priming significantly reduced the NaCl-induced accumulation of total phenolic content (TPC) in the roots (67.5%) (Fig. 5d). Compared with the controls, NaCl treatment stimulated the endogenous H_2_S level in the shoots (134.8%) and roots (188.6%) which was further boosted by NaHS priming to 252.8% and 693.5% in the shoots and roots, respectively, relative to the NaCl-stressed seedlings (Fig. 5e). NaHS pretreatment alone also increased the endogenous H_2_S level in the shoot (111.6%) and in the roots (180.3%) compared with the plants that received no NaCl stress (Fig. 5e). NaCl-induced elevation in the TT content of the sunflower shoots and roots was further increased by NaHS priming relative to seedlings grown under saline and non-saline conditions (Fig. 5f).

## Discussion

One of the most negative impacts of salinity stress is the reduction in plant growth and productivity despite the escalating demand for food all over the world [13, 36]. NaCl-induced growth reduction in sunflower seedlings observed in this study was most probably resulted from Na^+^ toxicity [36, 37], declined essential nutrient elements [38–40], reduced water absorption resulting from osmotic stress [41], and/or high ROS production [40, 42, 43]. These salinity-hazardous impacts adversely affect crop species by impairing various physiological, biochemical, and molecular processes which were reflected in growth and yield reduction [16, 40, 43, 44]. Our study showed that seed priming with NaHS significantly alleviated the deleterious effects of salinity on sunflower seedling growth, which is consistent with earlier studies by Sun and Luo [45] in cucumber, Chen et al. [46] in barley, Chen et al. [43] in *Cyclocarya paliurus*, Dawood et al. [39] in common bean, and Ding et al. [47] in wheat, who reported improved seed germination, biomass production, growth, and salinity tolerance of crop plants pretreated with NaHS under saline conditions. The ameliorative effect of H_2_S observed in the current work is most likely attributed to H_2_S impact on the retention of essential minerals (e.g., K^+^, Ca^2+^, Mg^2+^), decreased Na^+^ uptake and translocation that may effectively activate various events associated with stress adaptation and enhanced growth. Similarly, a strong correlation between salinity stress tolerance and crop ability to prevent NaCl-induced K^+^ leak from roots and retain low Na^+^ concentration by H_2_S application has been reported by other researchers in different crops [39, 48–51]. Additionally, H_2_S priming-stimulated ion homeostasis was possibly a major factor that participated in the improved salinity tolerance index of sunflower seedlings reported in this study, which agrees with previous works that demonstrate NaHS-enhanced salinity tolerance is associated with ion homeostasis in various crop species [46, 49–53].

Maintenance of ion homeostasis and enough nutrients are critically important for plants to preserve their structure and vital physiological activities under salinity stress [10]. Exposure of sunflower seedlings to 150 mM NaCl induced excessive Na^+^ accumulation and K^+^ insufficiency resulting in an increased Na^+^/K^+^ ratio, and reduced levels of Ca^2+^, Mg^2+^, and P. This disturbed ion homeostasis in response to NaCl exposure has been previously reported in other crops [38, 47, 51, 54]. The result could be attributed to the antagonistic impact of Na^+^ on K^+^ binding sites of the root plasma membranes [55], salinity-induced plasma membrane depolarization-initiated K^+^ leak [47, 56], the competitive uptake of Na^+^ and Cl^−^ with Ca^2+^, Mg^2+^, and P nutrients in the root plasma membranes [57], resulting in essential nutrient deficiency and elevated toxic ions. However, H_2_S priming enhanced the mineral content of sunflower seedlings and restored the nutrient pool, which is crucial for triggering several physiological and biochemical events related to stress adaptation. Ion homeostasis restoration induced by H_2_S treatment under salinity stress most probably is explained by H_2_S-improved maintenance of the plasma membrane integrity, thus preventing the K^+^ efflux and maintaining Ca^2+^ and other beneficial nutrients [52, 53, 58]. Another possible explanation might be due to H_2_S-enhanced K^+^ and Ca^2+^ levels that induce the plasma membrane ATPase activity providing the chemical potential gradient required for Na^+^/H^+^ antiport work [5, 46, 50, 59], thus accelerating K^+^ influx and Na^+^ efflux (reflected in lower Na^+^/K^+^ ratio obtained in this study) on one hand. On the other hand, ATPase activity possibly hyperpolarized the plasma membrane which also promoted the entry of K^+^ [5, 10, 60].

As SOS pathway is reported to be involved in regulating the ion levels in the cytosol [7, 61, 62], and *HaSOS2 *has been shown as a candidate gene to enhance salinity tolerance and as a central regulator of SOS pathway [14, 63], *HaSOS2* gene transcription in H_2_S primed and salinity stressed sunflower seedlings was determined. Supplementation of H_2_S significantly further induced upregulation of *HaSOS2* transcripts in salinity-stressed sunflower seedlings, which agrees with previously published works that indicate H_2_S upregulation of the expression of *HaSOS* genes triggered SOS-mediated Na^+^ efflux as an important strategy for maintenance of ionic homeostasis and crop salinity tolerance [49, 64–66]. Therefore, H_2_S-enhanced *HaSOS2* gene transcription might be an important strategy for the maintenance of ionic homeostasis and salinity tolerance in sunflower seedlings, and we suggest it as a biotechnological target for promoting crop tolerance to saline soil. NaCl-induced *HaSOS2* gene transcription most likely was not sufficient to reduce Na^+^ level and contribute to ion homeostasis under NaCl stress alone.

A growing body of evidence indicates that salinity imposition is associated with oxidative stress resulting from excessive ROS production [13, 16, 58, 67–70]. Research by Jiang et al. [58] demonstrated NaCl treatment triggered the accumulation of H_2_O_2_ that was associated with significant increments in EL and MDA levels in the leaves and roots of cucumber plants. Ahanger et al. [68] also illustrated reduced membrane stability in response to salinity-mediated accumulation of H_2_O_2_ in potato seedlings. Similarly, elevated oxidative stress indicators (MDA, H_2_O_2_) are reported in maize under a saline environment [67, 70, 71]. In the present study, NaCl stress increased EL, H_2_O_2_, and MDA overproduction in both sunflower shoots and roots, which was also confirmed by the impaired ROS scavenging capacity as evidenced by the dropped level of DPPH. Seed priming with H_2_S, however, successfully alleviated salinity-induced oxidative damage as proved by reduced levels of H_2_O_2_, MDA, and EL along with enhanced DPPH radical scavenging activity in both sunflower shoots and roots. Consistent with our results, NaHS pretreatment has mitigated salinity stress by lowering H_2_O_2_, MDA, and EL in rice [53], cucumber [58], *Malus hupehensis* [66, 72], and common bean [39]. Three different mechanisms are suggested to mediate H_2_S amelioration of NaCl-induced oxidative damage: first, H_2_S can interact directly with oxidants like H_2_O_2_, superoxide radical, the hydroxyl radical, and peroxynitrite, which effectively detoxified their deleterious impact [73]; second, H_2_S may induce higher levels of NO that acts as a scavenger of ROS [74] as well as H_2_S crosstalk with NO might modulate oxidative stress by inducing the antioxidant system [42]; third, H_2_S priming decreased the buildup of Na^+^, retained K^+^ and Ca^2+^ mediating ionic and redox homeostasis, which regulates ROS production [16, 62, 75]. In agreement with our assumption, Amooaghaie and Enteshari [76] reported that H_2_S application directly reacts with lipid radicals to prevent lipid oxidation, and/or H_2_S can indirectly amplify the activation of the antioxidant system to decrease salinity-induced oxidative damage.

One crucial mechanism evolved by plants to adapt to salinity stress includes the induction of enzymatic and non-enzymatic antioxidants, which are important for eliminating ROS and maintaining cellular redox potential [14, 77]. Under abiotic-induced oxidative stress, SOD scavenges O⋅ ^−^ into O_2_ and H_2_O_2_ as the first stage of the defense system, and the peroxidases act on the generated H_2_O_2_ reducing it to H_2_O. Further, APX, MDHAR, DHAR, and GR of the ascorbate–glutathione cycle act coordinately in eliminating H_2_O_2_ and regenerating AsA and GSH, which are crucial for promoting plant tolerance to abiotic stresses [78]. Although NaCl induced the activities of SOD, CAT, POD, APX, MDHAR, DHAR, and GR enzymes, elevated level of H_2_O_2_ was still obtained in the sunflower shoots and roots, suggesting H_2_O_2_ production exceeded ROS-scavenging capacity and might be considered a harmful impact of NaCl-stressed sunflower seedlings in absence of H_2_S; a similar response has been previously reported in rice by Mishra et al. [79]. NaHS-pretreated stressed sunflower seedlings, on the other hand, exhibited a significant reduction in oxidative stress-induced cellular injuries through an evident declined level of H_2_O_2_ concomitant with enhanced activities of the AsA-GSH cycle enzymes and increased content of GSH and AsA, indicative of H_2_S function was directly associated with the activation of AsA-GSH pathway [22]. In agreement, Lai et al. [52], Mostofa et al. [53], Dawood et al. [39], and Kumari et al. [22] have reported that H_2_S induction of the AsA-GSH cycle contributed to maintained redox status in various crop species. Moreover, to scavenge ROS directly, GSH detoxifies endobiotic substrates with the assistance of the GST enzyme [80]. The results of this research clearly showed that H_2_S boosted GST activity probably by promoting its biosynthesis through upregulating the *HaGST* gene expression, which might also maintain the cellular integrity and GST-mediated electrophile scavenging potential under salinity stress. Our proposal is supported by the data of Mostofa et al. [53], Ding et al. [48], and Dawood et al. [39] who reported NaHS-pretreatment significantly triggered the enhancement of GSH metabolizing enzymes by regulating their gene expression and activities, which results in scavenging of the oxidative damage induced by NaCl stress.

Besides enzymatic antioxidants, plants possess antioxidant compounds such as AsA, GSH, proline, glycine betaine, phenolics, trehalose, and α-tocopherols which work coordinately to scavenge ROS efficiently under stress [13, 78, 81]. H_2_S priming enhanced the overproduction of α-tocopherol content in salinity-stressed sunflower shoots, which may contribute to the enhancement of the antioxidant defense system and quick elimination of ROS. In support, α-tocopherol is involved in the direct scavenging of singlet oxygen (^1^O_2_) and hydrogen peroxide (H_2_O_2_) in tomato and wheat under abiotic stresses [82, 83]. Notably, α-tocopherol was not detected in sunflower roots in response to NaCl and/or H_2_S treatment. Presumably, α-tocopherol is synthesized and accumulated in the shoots because one of its main roles is to preserve the redox state in the chloroplasts and maintain the thylakoid membrane structure and function during plant development and response to stresses [84], while the main form of tocopherols prevailing in roots and seeds has been indicated to be γ-tocopherol, which can functionally substitute α-tocopherol [85, 86]. As for phenolic compounds, H_2_S pretreatment reduced the salinity-induced accumulation of phenols particularly in the sunflower roots; most likely H_2_S executed its antioxidant role via reestablishing redox homeostasis mainly by increasing the levels of AsA and GSH rather than phenols.

Although different kinds of environmental stresses caused differential dynamic changes in endogenous H_2_S metabolism, H_2_S application has been shown to greatly induce endogenous H_2_S production [39], which was also observed in this work. NaHS treatment significantly boosts NaCl-induced endogenous H_2_S and was associated with enhanced total thiol contents in both shoots and roots of sunflower seedlings. The elevation in the endogenous H_2_S level after stress treatment is indicative of H_2_S ability to be an important secondary messenger of stress detection, which in turn modulates plant physiological changes and downstream gene expressions [64]. As such, other studies reported salinity induction of increments in the endogenous H_2_S levels in strawberry [64], alfalfa [52], and common bean [39]. Enhancing TT content along with increasing endogenous H_2_S obtained in this study may be attributed to H_2_S-induced regulation of dynamic sulfur metabolism in plants, thus promoting the production of sulfur derivatives particularly GSH, as well as sulfur-containing proteins [66]. Also, H_2_S-mediated NO production and the interaction between them can generate new nitrosothiols, increasing TT content [87]. In accordance, the results of Chen et al. [88] showed increased internal contents of H_2_S, glutathione, cysteine, and TT in response to NaHS treatment in *Spinacia oleracea* seedlings.

## Conclusions

The present study provided a novel approach for unraveling the potential role of H_2_S in the alleviation of salinity-induced adversities in sunflower seedlings. H_2_S-mediated responses that confer salinity tolerance through two mechanisms: 1) maintained ion homeostasis by restricting the uptake of Na^+^, increasing K^+^ and Ca^2+^ contents as well as regulating the expression of *HaSOS2* gene, and 2) reduced oxidative damage, principally by regulating the AsA-GSH cycle and scavenging of peroxides through upregulation of *HaGST* gene along with GST activity stimulation, leading to declining ROS accumulation and consequently their cellular injuries. Therefore, H_2_S acts as a regulatory molecule activating these functional processes responsible for sunflower adaptive mechanisms and could be adopted as a crucial crop management strategy to combat saline conditions in future research. However, using environmentally friendly substances for exogenous applications in natural field conditions will be of great interest to a full understanding of salinity-resistant mechanisms and improving crop yield, and may support our claims of the results observed in the controlled environments.

## Materials and methods

Sunflower (*Helianthus annuus* L.) seeds (cv. Sakha 53) were obtained from the Agricultural Research Center, Giza, Egypt, and kept in the dark at 4 °C. Sunflower generally has been considered a sensitive plant to abiotic stresses (Tyagi et al*.,* 2018). For the experiment, the seeds were surface sterilized by immersion in 1% (w/v) sodium hypochlorite solution for 10 min and then rinsed thoroughly with distilled water. The sterilized sunflower seeds were divided into two groups at room temperature (25 ± 2 °C): the first group was primed with distilled water (control). The second group was primed for 2 h with NaHS (Sigma, USA) as the exogenous H_2_S donor at a concentration of 0.5 mM. The H_2_S concentration was used based on a trial experiment that exhibited the best results on sunflower germination and growth. The seeds were then dried on filter paper for approximately 24 h before germination. Ten seeds were directly sown in plastic pots (diameter 15 cm, height 30 cm) containing 1.5 kg of sieved air-dried clay soil and peat moss (peat moss: clay, 1:1 v/v). The seedlings were grouped into three replications of ten seedlings per replicate. The experiment was conducted using a completely randomized design using a controlled growth chamber (model V3-DM, Vision Scientific Company, Daejeon-Si, Korea) which was maintained at 27/18 °C day/night temperatures, a 50% relative humidity, a photosynthetic photon flux density (PPFD) of 400 μmol m^−2^ s^−1^, and a 14-h photoperiod. NaHS-pretreated and non-pretreated seedlings were irrigated every other day with tape water until seedling establishment for 14 d, and then they were exposed to two levels of NaCl (0 and 150 mM) for 7 d. Therefore, the following treatments of sunflower seedlings were established: (1) control, untreated and irrigated with tape water; (2) NaCl, untreated and irrigated with 150 mM NaCl solution; (3) NaHS, NaHS-priming and irrigated with tape water; (4) NaHS + NaCl, NaHS-priming and irrigated with 150 mM NaCl solution. The seedlings were watered every other day with 75 mL of the respective treatment. At the end of the experiment, 21-day-old seedlings were harvested and growth criteria, STI, and EL were measured. The remaining seedlings were immediately frozen in liquid nitrogen and stored at —80 °C for other physiological and biochemical analyses.

### Determination of growth parameters and STI

The plants were cut at the shoot base to give the shoots and roots, which were immediately weighed to determine their fresh weight (FW, g per shoot or root). The shoots and roots were then dried at 60 °C for 96 h to obtain the dry weight (DW, g per shoot or root). The STI was calculated as the total plant dry weight of salinity stress compared with the total plant dry weight of the control (0 salinity) based on the equation of Bağci et al. [89]:

$$\mathrm{STI }= (\mathrm{DW\ at\ Sx\ }/\mathrm{DW\ at\ S}1) \times 100$$

Where STI = salinity tolerance index, S1 = control, Sx = salinity treatment.

### Elemental analysis, Na^+^ uptake, and translocation

To determine the shoot and root mineral nutrient contents; Na^**+**^, K^**+**^, Ca^**2+**^, Mg^**2+**^, and P as well as the Na^**+**^/K^**+**^ ratio, dried samples (0.1 g) were ground and digested with an HNO_3_:HClO_4_ (5:1 v/v) mixture at 80°C until the yellow color disappeared. The contents of Na^**+**^, K^**+**^, Ca^**2+**^, and Mg^**2+**^ were measured using flame atomic absorption spectrophotometry (Savant AA, GBC, Australia). P was determined calorimetrically (HI 835200 Multiparameter Bench photometer, UK) according to the method of Jackson [90]. The results were expressed as µg of the metal per g DW of the sample. Sodium uptake at the sunflower root surface and ionic Na^+^ translocation from root to shoot were calculated according to Malik et al. [91] using the following equations:

$$\begin{array}{c}\mathrm{Na}^+\mathrm{ uptake }=\mathrm{ total\ }[\mathrm{Na}^+]\mathrm{\ in\ plant}/\mathrm{root\ dry\ weight}\\ \mathrm{Na}^+\mathrm{ translocation }= [\mathrm{Na}^+]\mathrm{\ in\ shoot}/[\mathrm{Na}^+]\mathrm{\ in\ root}\end{array}$$

### Determination of EL and the content of MDA

As salinity imposition induces excessive ROS generation resulting in oxidative stress, membrane integrity in response to salinity and H_2_S priming was evaluated in the leaves and roots by measuring the EL as described by Valentovic et al. [92] using the HANNA conductivity meter (HI8733, UK). The EL was determined according to the following equation:

$$\mathrm{EL\ }(\mathrm{\%}) = (\mathrm{L}1/\mathrm{L}2) \times 100$$

Where L1 refers to the electric conductivity (EC) of the outer de-ionized water where the leaves were soaked for 24 h at 25°C, and L2 is the EC of the outer de-ionized water after autoclaving at 120 °C for 20 min.

MDA was determined by the thiobarbituric acid (TBA)–based colorimetric method as described by Heath and Packer [93]. Half a gram of leaf sample was extracted in 3 mL of trichloroacetic acid (TCA) and centrifuged at 11,500 × g for 12 min. The supernatant (1 mL) was integrated with 4 mL of thiobarbituric acid (TBA) reagent (0.5% TBA + 20% TCA) in a 95 °C water bath for 30 min. After cooling, the density of the colored chromophore was observed using a colorimeter. The results were expressed as µmol MDA g^−1^ FW using the molar extinction coefficient of 155 mM^−1^ cm^−1^.

### Determination of H_2_O_2_ content

H_2_O_2_ was determined by the methods of Velikova et al. [94]. Leaf tissues (0.5 g) were homogenized in an ice bath with 5 mL 0.1% (w/v) trichloroacetic acid. The homogenate was centrifuged at 12,000 *g* for 15 min and 0.5 mL of the supernatant was added to 0.5 mL of 10 mM potassium phosphate buffer (pH 7.0) and 1 mL of 1 M KI. The absorbance of the supernatant was measured at 390 nm. The content of H_2_O_2_ was determined using a standard curve and expressed as µmol g^−1^ FW.

### DPPH radical scavenging assay

DPPH radical scavenging is a popular spectrophotometric method that has a wide application area and is used for determining the antioxidant capacity of various compounds. The activity of 2,2‐diphenyl‐1‐picrylhydrazyl (DPPH) radical scavenging was determined using the method of Hatano et al. [95]. The radical-scavenging activity was calculated as a percentage according to the equation:

$$\mathrm{DPPH\ radical}-\mathrm{scavenging }\ (\mathrm{\%}) = (\text{absorbance of the control }-\text{ the absorbance of the sample})/\text{absorbance of the control }\times 100$$

### Determination of antioxidant enzyme activities and nonenzymatic antioxidant contents

Fresh samples (0.5 g) were ground in liquid nitrogen and homogenized in Tris–HCl (100 mM, pH 8.0) extraction buffer containing EDTA (1 mM), DTT (5 mM), Triton X-100 (0.02%, v/v), and glycerol (10%, v/v). The resulting homogenates were centrifuged at 17,000 *g* for 20 min at 4 °C. The supernatants were used for the determination of enzyme activities. SOD (EC 1.15.1.1) activity was measured according to Marklund and Marklund [96]. CAT (EC 1.11.1.6) activity was assayed following the method of Aebi [97]. POD (EC 1.11.1.7) activity was determined based on the method of Shannon et al. [98]. DHAR (EC 1.8.5.1) activity was measured using the method of De Tullio et al. [99]. MDHAR (EC 1.6.5.4) activity was assayed according to the method of Hossain and Asada [100]. GR (EC 1.6.4.2) was assayed by the method of Goldberg and Spooner [101] using kits (Biodiagnostics, Egypt). GST (EC 2.5.1.18) was carried out according to Habig and Jakoby [102]. APX (EC 1.11.1.11) activity was measured by the method of Chen and Asada [103].

Non-enzymatic antioxidants including TPC and AsA were determined according to Makkar et al*.* [104] and Mukherjee and Choudhuri [105], respectively. GSH was determined according to the modified method of Vlachaki and Meyn [106]. The content of α-tocopherol was quantified by using high-performance liquid chromatography (HPLC; instrument E-Chrom Tech, LC 1620, USA) with electrochemical detection according to Desai [107]. Extraction from the samples was performed with 1 mL of methanol and 4 mL of hexane. After the samples were centrifuged at 1500 *g* for 10 min, the hexane phase was removed and evaporated to dryness under N_2_. Samples were dissolved in methanol: ethanol (1:1, v/v) and injected for HPLC analysis. *d,l-α* T from synthetic phytol (Sigma, USA) was used as standard.

### Determination of endogenous H_2_S and TT contents

The endogenous H_2_S content of sunflower leaves and roots was determined using 2 g FW each, which was extracted and homogenized in 20 mM Tris–HCl buffer (pH 8.0) containing 10 mM EDTA and 20 mM zinc acetate to stabilize H_2_S. After centrifugation at 15,000 *g* for 15 min at 4 °C, 1 mL of 30 mM FeCl_3_ dissolved in 1.2 M HCl and 1 mL of 20 mM DMPD dissolved in 7.2 M HCl mix were added. The test mixture was incubated at room temperature for 15 min, and the absorbance was read at 670 nm. The content of H_2_S was determined and expressed as μg g^−1^ FW from a standard curve of an appropriate donor of NaHS based on the method of Li et al. [108].

Total thiols were estimated as described by Nagalakshmi and Prasad [109]. Fresh shoots (0.5 g) were homogenized in 20 mM ascorbate buffer containing 20 mM EDTA, and the homogenates were centrifuged at 12,000 *g* for 20 min at 4°C. Aliquots (0.5 mL) of the supernatants were mixed with 2.4 mL of 200 mM Tris–HCl buffer (pH 8.2) and 0.1 mL of 10 mM DTNB. The color was allowed to develop for 15 min, and then the absorbance was measured at 412 nm. The TT content was calculated and expressed as μg g^−1^ FW via a standard curve prepared with known concentrations of GSH.

### RNA extraction and Quantitative Real-Time PCR (qRT-PCR)

Total RNA was extracted from 30 mg of leaf FW for each treatment using a Gene JET™ RNA purification Kit (Thermo Fisher Scientific, MA, USA). One μg of total RNA was reverse transcribed into cDNA using the Revert Aid First Strand cDNA Synthesis Kit (Thermo Fisher Scientific, MA, USA). Primer sequences for the *HaSOS2* gene reactions were 5′-AATTTGGATGATATTCGTGCAGTTTTTG-3′ and 5′-TTAACATTTAAATGGAATTGACC-3′/ synthesized from Gene link, USA, as described by Halfter et al. [110].

The primer pairs used for the *HaGST* gene were 5′-TTGTGGAGAGGATCAGAGG-3′ and 5′-TTTAGCCGAAAAGGGTATT-3′ as described by Ma et al. [111]. Actin sequence from sunflower 5′-AGGGCGGTCTTTCCAAGTAT -3′ and 5′-ACATACATGGCGGGAACATT -3′ was used as a reference gene to normalize the relative transcription and to minimize different copy numbers of cDNA templates. PCR amplification specificity was verified using melting curve analysis and data were analyzed using the 2^−ΔΔCt^ method according to Livak and Schmittgen [112] after normalizing to the expression of each actin gene.

### Statistical analysis

The results were subjected to a one-way analysis of variance (ANOVA) using the software package SPSS v20.0 (SPSS Inc., Chicago, USA). The means of different treatments were compared using Duncan’s multiple range test at a significance level of 5% (P ≤ 0.05).

## Data Availability

The datasets analyzed during the study can be obtained from the corresponding author on request.

## Abbreviations

APX: Ascorbate peroxidase
AsA-GSH cycle: Ascorbate-glutathione cycle
CAT: Catalase
DHAR: Dehydroascorbate reductase
DPPH: 2,2‐Diphenyl‐1‐picrylhydrazyl
EC: Electric conductivity
EL: Electrolyte leakage
GR: Glutathione reductase
GST: Glutathione S-transferase
HPLC: High-performance liquid chromatography
MDA: Malondialdehyde
MDHAR: Monodehydroascorbate reductase
POD: Peroxidase
qRT-PCR: Quantitative Real-Time PCR
ROS: Reactive oxygen species
SOD: Superoxide dismutase
SOS: Salt Overly Sensitive
STI: Salt tolerance index
TPC: Total phenolic content
TT: Total thiol
